# Aberrant Histone Methylation in Patients with Graves' Disease

**DOI:** 10.1155/2019/1454617

**Published:** 2019-06-24

**Authors:** Ni Yan, Kaida Mu, Xiao-fei An, Ling Li, Qiu Qin, Rong-hua Song, Qiu-ming Yao, Xiao-qing Shao, Jin-an Zhang

**Affiliations:** ^1^Department of Endocrinology, Shaanxi Provincial People's Hospital, No. 256 West Youyi Road, Beilin District, Xi'an 710068, China; ^2^Department of Endocrinology, Shanghai University of Medicine & Health Sciences Affiliated Zhoupu Hospital, No. 1500 Zhouyuan Road, Pudong New District, Shanghai 201318, China; ^3^Department of Endocrinology, Jinshan Hospital of Fudan University, No. 1508 Longyang Road, Jinshan District, Shanghai 201508, China; ^4^Department of Endocrinology, The Affiliated Hospital of Nanjing University of Chinese Medicine, No. 138 Xianlin Road, Qixia District, Nanjing 210023, China

## Abstract

**Background:**

Graves' disease (GD) is an organ-specific autoimmune disease. Accumulated data have indicated that aberrant epigenetic modifications are associated with many autoimmune disorders. However, it remains unknown whether histone methylation plays a role in the pathogenesis of GD. In the present study, we aimed to assess histone modification patterns in peripheral blood mononuclear cells (PBMCs) from GD patients. The rate (degree) of H3K4 and H3K9 methylation and the expressions of histone-modifying genes were investigated.

**Methods:**

A total of 68 GD patients and 32 healthy controls were enrolled in this study. Global histone H3K4/H3K9 methylation of PBMCs was evaluated by the EpiQuik™ global histone H3K4/H3K9 methylation assay kit. The expressions of histone methyltransferases (HMTs) and histone demethylases (HDMs) at the mRNA level were determined by real-time quantitative polymerase chain reaction.

**Results:**

Global histone H3K9 methylation in PBMCs of GD patients was significantly decreased compared with that in the healthy controls (P=0.007). The expressions of HMTs (SUV39H1 and SUV39H2) at the mRNA level were significantly decreased in PBMCs from GD patients compared with healthy controls (P<0.001), whereas the SETD1A expression at the mRNA level was significantly increased in GD patients compared with healthy controls (P=0.004). In addition, the expressions of HDMs, including JHDM2A and JMJD2A, at the mRNA level were significantly increased in GD patients compared with healthy controls (P<0.001; P=0.007). Moreover, the mRNA expression levels of JARID1A and LSD1 did not significantly differ in GD patients and healthy controls (P>0.05).

**Conclusions:**

These findings firstly suggested that the histone methylation was aberrant in PBMCs of GD patients, which could be possibly attributed to the deregulation of epigenetic modifier genes. Abnormal histone methylation modification may be involved in the pathogenesis of GD.

## 1. Introduction

Graves' disease (GD) is the most common autoimmune disease, affecting 0.5% of the total population, and it represents 50-80% of the cases of hyperthyroidism [1]. Its typical manifestations include the unique association with thyrotoxicosis, goiter, and ophthalmopathy. As for the pathogenesis, GD is characterized by lymphocyte infiltration in thyroid tissue, leading to production of thyroid-stimulating hormone receptor (TSHR) antibody (TSAb), which in turn increases synthesis and release of thyroid hormones (hyperthyroidism) and induces hypertrophy of thyroid follicular cells (goiter). Ophthalmopathy, the most common extrathyroidal feature of GD, is clinically present in about 50% of patients [2].

As a multifactorial or so-called “complex” disease, GD is caused by the confluence of genetic susceptibility and environmental factors, leading to loss of immune self-tolerance at central and peripheral levels [3]. According to twin studies, genetic factors account for approximately 80% of the risk for GD development [4]. In addition to the MHC class II genes, we and other investigators have found that several other gene loci are associated with GD, including immune-regulatory (CD40, CTLA-4, PTPN22, FOXP3, and CD25) and thyroid-specific genes (thyroglobulin and TSHR) [5–8]. Among nongenetic factors, iodine, infection, psychological stress, gender, smoking, vitamin D, and selenium deficiency may contribute to the occurrence and progression of the disease [9]. Considerable progress has been made to enhance our understanding of the etiology of GD. However, it remains largely unexplored how the autoimmune response is triggered. Increasing evidence suggests that epigenetic modifications bridge the gap between genetic susceptibility and the environment, thus triggering GD. Epigenetics refers to the system that governs the long-term stable regulation of gene expression profile that does not involve changes in gene sequences [10]. The term of epigenetic effect generally suggests noncoding effects on gene expression and function, but such effects are mitotically stable and can last for a long time. There are different epigenetic mechanisms, including DNA methylation, histone modification (usually acetylation, de-acetylation, methylation, and phosphorylation), nucleosome positioning, RNA interference (RNAi), miRNA, and small interfering RNA (siRNA) [11–13].

Histone modification plays an important role in transcriptional regulation, DNA repair, DNA replication, and chromosome condensation [14, 15]. Histone methyltransferases (HMTs) and histone demethylases (HDMs) are enzymes that catalyze the addition and removal of histones methyl groups at lysine and arginine residues [16]. Lysine residues in histone H3 can be mono-, di-, or trimethylated. Previous studies have demonstrated that methylation of histone H3 at lysine 9 (H3K9) and H3K27 is associated with transcriptional repression, whereas methylation at H3K4, H3K36, and H3K79 is associated with transcriptional activation [16]. Furthermore, H3K4 methylation is associated with euchromatin function, and H3K27 methylation is involved in X-chromosome inactivation [17].

Epigenetic mechanisms are a window, through which we can understand the possible mechanisms involved in the pathogenesis of complex diseases, such as autoimmune diseases. Recently, our studies have been the first to find that the histone acetylation and DNA methylation are aberrant in peripheral blood mononuclear cells (PBMCs) from GD patients [18, 19]. Growing evidence also supports that histone methylation has been involved in the development of other autoimmune diseases, such as rheumatoid arthritis [20, 21], systemic lupus erythematosus [22, 23], systemic sclerosis (SSc) [24], type 1 diabetes [25, 26], psoriasis [27], Henoch–Schönlein purpura [28], immune thrombocytopenia [29], and pemphigus vulgaris (PV) [30]. To date, the state of histone methylation has not been explored in GD. In this study, we, for the first time, investigated whether the histone methylation modification pattern was altered in GD patients.

## 2. Materials and Methods

### 2.1. Subjects

In the present study, 68 subjects (50 males and 18 females), who were primarily diagnosed with GD (at a mean age of 34.83±9.59 years) and not given any medication, were enrolled from the Outpatient Clinic of the Department of Endocrinology, Jinshan Hospital of Fudan University, China. GD was diagnosed based on clinical manifestations and laboratory examinations. The primary clinical manifestations for GD included weight loss despite a hearty appetite, heat intolerance, palpitations, and thyrotoxicosis-induced tremor. Most patients had a diffuse goiter, and GD in a few patients was accompanied by ophthalmopathy and dermopathy. The laboratory diagnostic criteria consisted of hyperthyroidism (elevated thyroid hormone and suppressed TSH) and autoimmune biomarkers, including positive serum antibodies to TSH-receptor (TRAb), with or without anti-thyroid peroxidase antibody (TPO-Ab) or anti-thyroglobulin (Tg-Ab).

A total of 32 healthy controls (35.25±7.43 years, 10 males and 22 females) with no family history of thyroid diseases or other autoimmune diseases were recruited from the Health Care Center of the same hospital.

This study was approved by the Human Ethics Committee of Jinshan Hospital of Fudan University, and written informed consent was obtained from all the participants.

### 2.2. Isolation of PBMCs

PBMCs were isolated from 10 mL venous peripheral blood preserved in heparin from each subject by density gradient centrifugation using Ficoll-Hypaque media (Cat. LTS1077, Tianjin Haoyang Biological Manufacturer Co., Tianjin, China) and stored at −80°C until further analysis.

### 2.3. Extraction of Total Histone and Determination of Protein Concentration

Total histone was extracted from PBMCs using the EpiQuik™ total histone extraction kit (Cat. P-3017-96, Epigentek Group Inc., NY, USA) according to the manufacturer's instructions. Briefly, every 1 × 10^6^ cells were lysed with 10 *μ*L lysis buffer, mixed with three volumes of extraction buffer/glycerol solution by vortexing, and incubated on ice for 5 min. After centrifugation at 12,000 rpm for 5 min at 4°C, the supernatant was collected into a 1.5-mL tube, mixed with 100% TCA solution to a final concentration of 25%, and incubated on ice for 30 min to precipitate proteins. The pellet was collected by centrifugation at 12,000 rpm for 10 min at 4°C, washed twice with acetone, and dissolved in 10 *μ*L of water per amount of pellet extracted from 1 × 10^6^ cells. The protein concentration was determined using Beckman Coulter DU 730 Nucleic Acid/Protein Analyzer, and the extracted histone protein samples were aliquoted and stored at −80°C.

### 2.4. Measurement of Global Histone H3K4/H3K9 Methylation

Global histone H3K4/H3K9 methylation was assessed using the EpiQuik™ global histone H3K4/H3K9 methylation assay kit (Cat. P-3017-96/P-3018-96, Epigentek Group Inc., NY, USA) following the manufacturer's instructions. Briefly, histone proteins (1-2 *μ*g) were added to the strip wells. Methylated histone H3K4/H3K9 was detected with a high-affinity antibody, and the ratios and amounts of methylated histone H3K4/H3K9 were determined with a horseradish peroxidase-conjugated secondary antibody using a color development system. Absorbance was measured at a wavelength of 450 nm.

### 2.5. Nucleic Extraction

PBMCs were resuspended in 1 × prelysis buffer with gentle stirring. Cell pellet was collected by centrifugation at 1,000 rpm for 5 min. After being washed once with 10 mL of PBS, cell pellets were resuspended in 100 *μ*L of diluted lysis buffer/1 × 10^6^ cells and incubated on ice for 5 min. The supernatant was collected by centrifugation at 12,000 rpm for 30 s as the nucleic extraction.

### 2.6. RNA Isolation, cDNA Synthesis, and Real-Time Quantitative Reverse Transcription PCR (qRT-PCR)

Total RNA was extracted from PBMCs using TRIzol reagent (Cat. 10296-028, Invitrogen, USA) according to the manufacturer's instructions. RNA quality and purity were determined using NanoDrop® ND-1000 (Thermo Scientific, USA). Samples with OD_260nm_/OD_280nm_ between 1.0 and 2.0 were used. A total of 1 *μ*g of RNA was reversely transcribed into cDNA using a reverse transcription kit (Cat. DRR037A, Takara, Japan) at 37°C for 15 min followed by 85°C for 5 s. The expressions of target genes at the mRNA level were assessed using an ABI PRISM 7300 (Applied Biosystems, Foster City, CA, USA) and SYBR Premix Taq (Cat. RR820A, Takara, Japan).  Table 1 lists the sequences of primers.

The PCR reaction was performed in a 10 *μ*L reaction system consisting of 5 *μ*L of 2× SYBR Premix Ex Taq II, 1 *μ*L of 10 *μ*M primer pair, and 50 ng of cDNA. Briefly, after an initial denaturation step at 95°C for 30 s, the amplifications were carried out with 40 cycles at a melting temperature of 95°C for 5 s and an annealing temperature of 60°C for 31s. *β*-Actin was also amplified as an endogenous control to confirm that equal amounts of total RNA were added from each sample and to normalize the amount of total RNA. All PCR reactions were performed in triplicate. The mean value of the replicates for each sample was calculated and expressed as a cycle threshold (Ct) value. The relative expression of each target gene was determined using the 2^−ΔΔ ct^ method.

### 2.7. Statistical Analysis

All data are expressed as mean±SD (mean ± standard deviation). Statistical significance between different groups was determined using nonparametric Mann-Whitney U test. The Spearman rank correlation was used to analyze the correlation among the expressions of biomarkers and clinical stages. Statistical analysis was performed using SPSS 17.0 (http://www-01.ibm.com/software/analytics/spss/). P < 0.05 was considered as statistically significant.

## 3. Results

### 3.1. Global Hypomethylation of Histone H3K9 in PBMCs of GD Patients

**Figure 1:**
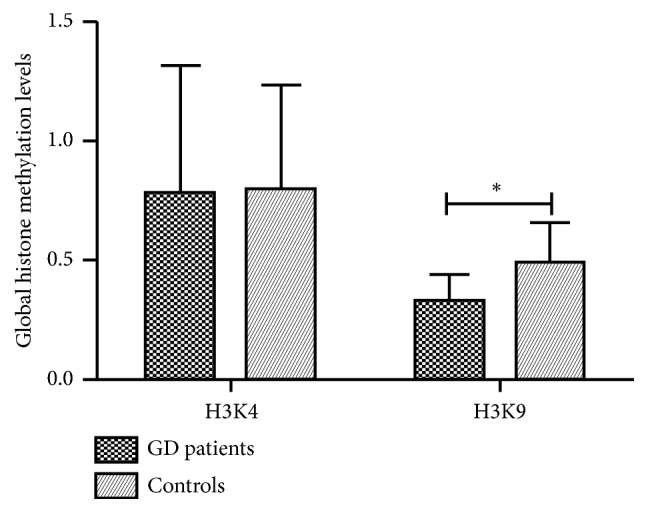
Global histone methylation levels in PBMCs from patients of GD and healthy controls. Mean global histone H3K9 methylation level was significantly decreased in PBMCs from GD patients in contrast to healthy controls (*∗*, P=0.007).

In the present study, the level of histone methylation was evaluated in PBMCs from GD patients using ELISA. The results demonstrated that the global histone H3K9 methylation was significantly downregulated in PBMCs of GD patients compared with normal controls (0.3274±0.1098 vs. 0.4910±0.1671, P=0.007, Figure 1). However, the global H3K4 methylation was decreased in GD patients despite having no statistical significance (0.7764±0.5391 vs. 0.7998±0.4371, P> 0.05).

### 3.2. Expressions of Histone-Modifying Genes in PBMCs of GD Patients

**Figure 2:**
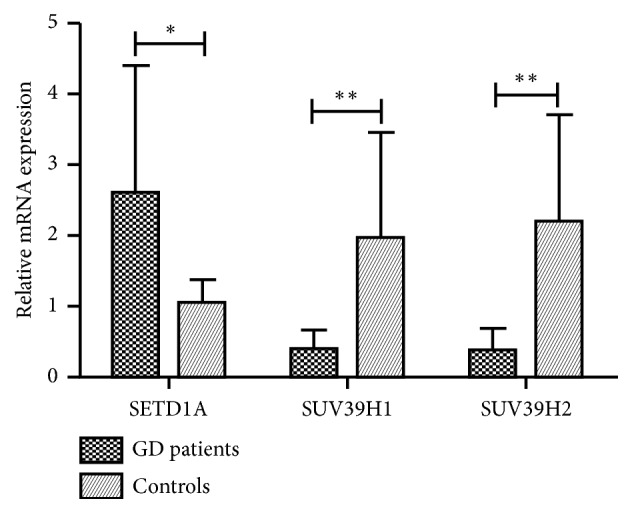
Relative mRNA levels of HMTs in PBMCs of patients with GD and healthy controls. Results represent mean ± SD expression levels normalized to *β*-actin. SUV39H1 and SUV39H2 mRNA expressions were significantly decreased (*∗∗*P<0.001), whereas the expression of SETD1A mRNA was higher in GD patients (*∗*P=0.004).

**Figure 3:**
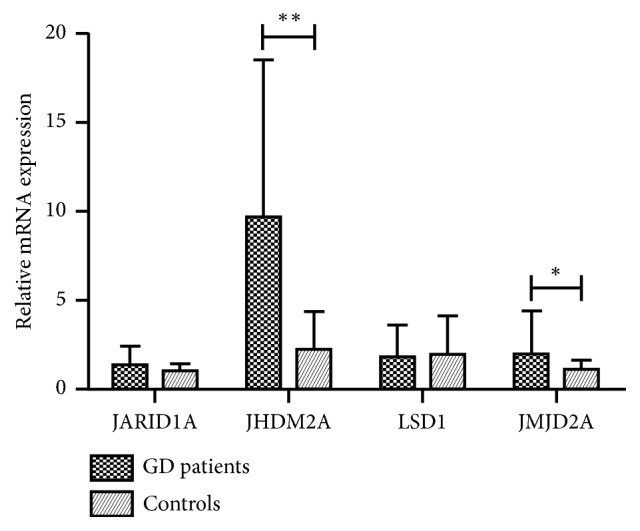
Relative mRNA levels of HDMs in PBMCs of patients with GD and healthy controls. Results represent mean ± SD expression levels normalized to *β*-actin. JHDM2A and JMJD2A were significantly increased in GD patients (*∗∗*P<0.001; *∗*P=0.007).

To further investigate the association between aberrant histone modulation and the expressions of chromatin modifier genes in PBMCs of GD patients, we detected the expressions of histone methylation modifier genes, including HMTs and HDMs, by qRT-PCR in 68 GD patients and 32 healthy controls. Figures 2 and 3 show the differential expressions of histone-modifying genes. For the HMT group, the expressions of SUV39H1 and SUV39H2 mRNAs were significantly decreased in PBMCs from GD patients compared with healthy controls (0.385±0.279 vs. 1.969±1.486 for SUV39H1, P<0.001; 0.379±0.301 vs. 2.192±1.509 for SUV39H2, P<0.001, Figure 2), whereas the expression of SETD1A mRNA was higher in GD patients (2.593±1.800 vs. 1.051±0.319, P=0.004, Figure 2). In addition, the expressions of HDMs, including JHDM2A and JMJD2A, were significantly increased in GD patients compared with healthy controls (9.696±8.849 vs. 2.234±2.175 for JHDM2A P<0.001; 1.969±2.428 vs. 1.117±0.527 for JMJD2A, P=0.007, Figure 3). Moreover, there were no significant differences between GD patients and healthy controls in the expressions of JARID1A and LSD1 (1.324±1.096 vs. 1.070±0.390 for JARID1A and 1.812±1.810 vs. 1.972±2.154 for LSD1, P>0.05, Figure 3).

Furthermore, the correlation analysis indicated that changes in methylation of global H3K9 were not correlated with the expressions of SUV39H21 and SUV39H2 (P>0.05). In addition, the histone H3K9 methylation was not correlated with laboratory indicators, including FT3, FT4, TSH, and TRAb (P>0.05).

## 4. Discussion

As an organ-specific autoimmune disease, GD is usually characterized by the presence of circulating autoantibodies that bind to TSHR on thyrocytes and mimic the effects of TSH, resulting in hyperthyroidism and goiter [2]. Nevertheless, considerable progress has been made in further understanding the genetic contribution to thyroid autoimmunity in GD. However, the mechanisms by which gene variants interact with environmental factors to cause GD remain largely unexplored. It is postulated that interactions of susceptibility genes with certain environmental factors trigger the onset of GD through epigenetic effects. The clinical manifestations of GD are likely to be the result of complex interactions between environmental and genetic susceptibility factors. Recent data have suggested that epigenetic mechanisms, including DNA methylation as well as histone acetylation, deacetylation, and methylation, may trigger gene environment interactions in complex diseases [12, 13]. Epigenetic changes have been shown to play a role in the etiology of autoimmune diseases, including type 1 diabetes, systemic lupus, and rheumatoid arthritis. It has been reported that IFN-*α* induces alterations in thyroglobulin gene expression through epigenetic changes in histone modifications [31]. Abnormal histone deacetylation has also been identified in patients with resistance to thyroid hormone (RTH) [32]. Also research on histone tail modifications is mainly available from GD patients. We are the first to demonstrate that histone H4 hypoacetylation is accompanied by upregulated expressions of HDAC1 and HDAC2 at the mRNA level in GD [18]. It also has been shown that impaired expressions of noncoding RNAs, particularly microRNAs (miRNAs), were identified in Hashimoto's thyroiditis (HT) individuals such as miR-142-5p, miR-142-3p, and miR-146a, which showed high expression in HT thyroid gland [33].

Previous study has revealed direct functional links between histone acetylation and methylation [34]. They synergistically regulate the chromatin structure critical for transcription activity [35]. In the present study, we identified a significantly decreased methylation of global histone H3K9 in PBMCs of GD patients, although no significant difference in global histone H3K4 methylation was observed. Similar to our results, global histone H3K9 hypomethylation has been observed in B cells from SSc patients, and there is no difference in the global H3K4 methylation between SSc patients and controls [24]. Global histone H3K9 hypomethylation in CD4^+^ T cells has been reported in patients with active immune thrombocytopenia (ITP) compared with ITP patients in remission and healthy controls, while the global histone H3K4 methylation is not significantly different between ITP patients and healthy controls [29]. In another study, the global histone H3K4 hypermethylation is observed in PBMCs from patients with Henoch–Schönlein purpura, while the global histone H3K9 methylation is not changed [28].

H3K4 hypomethylation is observed in PBMCs of patients with alopecia areata (AA), but there is no significant difference in H3K9 methylation between AA patients and healthy controls [36]. By combining the above-mentioned results with our findings, we deduce that the aberrant histone modification might play an important role in the pathogenesis of many autoimmune disorders, including GD.

Histone methylation is catalyzed by various histone-modifying enzymes, which add or remove methyl (HMTs and HDMs) groups to/from target histone. In this study, we found that HMTs, such as SUV39H1 and SUV39H2, were downregulated in PBMCs from GD patients, while SETD1A was upregulated. Moreover, HDMs (JHDM2A and JMJD2A) were upregulated in PBMCs from GD patients. All these results were consistent with the observed hypomethylation of H3K9.

SUV39H1 and SUV39H2 suppressors of variegation 3-9 homolog family move to the centromeres during mitosis and function as HMTs, methylating Lys-9 of histone H3 [37]. The degree of H3 Lys9 methylation by different methyltransferases can explain the dissimilar phenotypes observed in the transgenic mice [38]. Some studies have revealed that Su (var) 3-9 mainly controls H3K9 dimethylation and trimethylation in the pericentric heterochromatin [39]. It has been suggested that SUV39H1 and SUV39H2 are responsible for the majority of H3K9 trimethylation. In agreement with our results, SUV39H2 is significantly reduced and JHDM2A is increased at both mRNA and protein levels in B cells from SSc patients [24]. In PV, the SUV39H2 expression at the mRNA level is significantly increased, while the SUV39H1 expression at the mRNA level is significantly decreased in PBMCs from PV samples compared with controls, and the SETD1A expression is not significantly changed [30]. Besides, the SETD1A expression at the mRNA level is significantly increased in psoriatic patients [27]. In addition, the expression of SUV39H2 is significantly downregulated in active ITP patients compared with ITP patients in remission and healthy controls. There is no difference in the expression of SUV39H1 between ITP patients and controls [29]. However, the expressions of SUV39H1, SUV39H2, and SETD1A at the mRNA level in AA patients are not significantly different from those in healthy controls [36]. We also observed increased expressions of HDMs, such as JHDM2A and JMJD2A, in GD patients. These results were consistent with Wang's finding that the expressions of JHDM2A at the mRNA and protein levels are significantly increased in SSc B cells of AA patients [36]. This discrepancy may be attributed to the specific pathogenesis for a particular autoimmune disease although they somehow share the similar mechanisms.

We showed that H3K9 methylation and differential expression of HMTs and HDMs were not correlated with laboratory indicators, including FT3, FT4, TSH, and TRAb. These results might be caused by the limited number of the samples or other unknown factors. The measure of the expression at the mRNA level for an enzyme, by itself, does not demonstrate a functional role.

## 5. Conclusions

Taken together, our present study demonstrated aberrant histone methylation and expressions of chromatin modifier genes in PBMCs of GD patients. These results provide novel insights into the pathogenesis of GD and lay the foundation for understanding the involvement of epigenetic factors in promoting GD. Further studies are necessary to reveal the specific role of abnormal histone methylation modification in GD.

## Figures and Tables

**Table 1:** Primer sequences used for RT-PCR.

Gene	Primer	Sequence (5′ → 3′)
SETD1A	Forward	GCCACGCAGTGAGTTTGA

	Reverse	ACCCAGTGAGTGTCGTTGAG

JARID1A	Forward	CCGTCTTTGAGCCGAGTTG

	Reverse	GGACTCTTGGAGTGAAACGAAA

SUV39H1	Forward	CCTGCACAAGTTTGCCTACA

	Reverse	AGTGCGGAAGATGCAGAGAT

SUV39H2	Forward	ATCCCACCTGGTACTCCCATCT

	Reverse	GCAAAGCGAATACTGTGTGCC

JHDM2A	Forward	GTGCTCACGCTCGGAGAAA

	Reverse	AAACAGCTCGAATGGTCCCG

LSD1	Forward	TTCTGGAGGGTATGGAGACG

	Reverse	ACCTTCTGGGTCTGTTGTGG

JMJD2A	Forward	AGAGTTCCGCAAGATAGCCAA

	Reverse	AGTCCAGGATTGTTCTCAGCC

EZh2	Forward	ACATCCTGACTTCTGTGAG

	Reverse	GGAGACCAAGAATACATTA

*β*-Actin	Forward	CATTGCCGACAGGATGCAG

	Reverse	CTCGTCATACTCCTGCTTGCTG

## Data Availability

The data used to support the findings of this study are available from the corresponding author upon request.
